# Society of Physicians and Surgeons

**Published:** 1877-10

**Authors:** F. M. Hall


### SOCIETY OF PHYSICIANS AND SURGEONS.

(Reported by F. M. Hall, M. D.)

### Regular Meeting, September 10th, 1877.

The President, Prof. Wm. A. Byford, in the chair.

The society met, for the first time, this evening, in the rooms of the Chicago Medical Press Association (188 South Clark street), which had been kindly tendered them by the association for their meetings. The members seem to be well pleased with their accommodations, and fully appreciate the courtesy shown them.

After the meeting had been called to order by the President, the minutes of the preceding meeting were read by the Secretary, and accepted by the society.

John H. Rauch, M. D., President of the Illinois State Board of Health, having been proposed for membership at a previous meeting, the Secretary was instructed to cast the vote of the society in his favor.

As there was no further business of special importance before the society, an informal discussion of the use of eucalyptus followed.

Dr. Bevan had used it beneficially in cases of debility and prostration, accompanied by nervous excitability. He considered it very similar to valerian in its action. It was tonic, stimulant and slightly anodyne.

Dr. Byford agreed with Dr. Bevan in regard to its action. He had used it where there was great debility (accompanying uterine disease), with violent sub-occipital headache, and pain following the course of the spinal column, culminating in convulsions, and was well pleased with its effects.

Dr. Bridge said the elixir of eucalyptus would completely cover the disagreeable taste of quinine.

The proportions to be used are as follows : gr. j. to the ʒj. of the elixir. In speaking of the different preparations covering the taste of quinine, Dr. Merriman mentioned glycyrrhizin, which is the flavoring extract of glycyrrhiza: The glycyrrhizin (3 or 4 grains) should be taken into the mouth and allowed to dissolve until you obtain the full taste of the licorice. The quinine should then be taken into the mouth and immediately swallowed.

The meeting then adjourned.

